# Supplementary material for: Effects of Genotype and Modified Atmosphere Packaging on the Quality of Fresh-Cut Melons
Source: Foods. 2024 Jan 13;13(2):256. doi: 10.3390/foods13020256 (PMC10815012; doi:10.3390/foods13020256)
Supplement: Supplementary file 1 [file foods-13-00256-s001.zip › foods-2778913-supplementary.pdf]

Table 1. Effect of genotype, packaging conditions and storage duration on the soluble solids content (SSC, %) in fresh-cut melons

		Cantalupensis melon			*Inodorus* melon		
MA type	Perforation	Initial	7 days	14 days	Initial	7 days	15 days
Passive	Non-perforated	10.00 ± 0.77	9.65 ± 1.27	9.80 ± 0.30	10.56 ± 0.88	10.98 ± 0.89	10.37 ± 2.09
	2 macro-holes		9.70 ± 0.98	9.67 ± 0.58		10.80 ± 0.50	10.85 ±2 .65
	1 micro-hole		9.30 ± 0.98	10.05 ± 0.49		10.77 ± 0.83	10.05 ± 0.69
	2 micro-holes		8.90 ± 0.50	9.80 ± 0.60		10.73 ± 0.72	10.70 ± 2.35
Active	Non-perforated	10.00 ± 0.77	9.00 ± 1.76	9.30 ± 0.30	10.56 ± 0.88	11.00 ± 0.59	11.40 ± 2.55
	1 pinhole		9.05 ± 0.69	9.97 ± 0.53		10.60 ± 0.46	9.25 ± 0.88
	1 micro-hole		10.00 ± 0.80	9.35 ± 0.69		10.77 ± 0.47	9.45 ± 0.88
	2 micro-holes		9.30 ± 0.98	9.40 ± 0.60		10.77 ± 0.64	10.67 ± 2.00

Table 2. Effect of genotype, packaging conditions and storage duration on the firmness (N) of fresh-cut melons

		Cantalupensis melon			*Inodorus* melon		
MA type	Perforation	Initial	7 days	14 days	Initial	7 days	15 days
Passive	Non-perforated	7.53 ± 0.70	6.33 ± 0.67	6.58 ± 1.38	8.89 ± 0.55	8.60 ± 0.80	8.61 ± 0.77
	2 macro-holes		6.55 ± 0.59	5.54 ± 1.64		9.37 ± 1.99	7.97 ± 1.53
	1 micro-hole		6.73 ± 1.14	7.47 ± 0.82		8.49 ± 0.72	7.83 ± 1.36
	2 micro-holes		6.47 ± 0.57	6.00 ± 0.60		9.20 ± 0.82	7.38 ± 0.79
Active	Non-perforated	7.53 ± 0.70	6.47 ± 0.64	6.69 ± 1.49	8.89 ± 0.55	8.97 ± 1.28	9.08 ± 0.84
	1 pinhole		6.40 ± 1.11	6.47 ± 1.60		7.93 ± 0.96	7.85 ± 1.37
	1 micro-hole		6.40 ± 1.08	8.03 ± 1.05		8.48 ± 1.12	8.42 ± 0.97
	2 micro-holes		6.75 ± 0.57	6.07 ± 0.35		8.61 ± 0.96	7.71 ± 0.94
